# Quantum Dot-Sensitised Estrogen Receptor-α-Based Biosensor for 17β-Estradiol

**DOI:** 10.3390/bios13020242

**Published:** 2023-02-08

**Authors:** Abongile N. Jijana, Usisipho Feleni, Peter M. Ndangili, Mawethu Bilibana, Rachel F. Ajayi, Emmanuel I. Iwuoha

**Affiliations:** 1 Nanotechnology Innovation Centre, Advanced Materials Division, Mintek, Private Bag X3015, Randburg, Johannesburg 2125, South Africa; 2 SensorLab (University of the Western Cape Sensor Laboratories), 4th Floor Chemical Sciences Building, University of the Western Cape, Private Bag X17, Bellville, Cape Town 7535, South Africa; 3 Institute for Nanotechnology and Water Sustainability (iNanoWS), College of Science, Engineering and Technology, University of South Africa, Florida Campus, P/Bag X6, Florida, Roodepoort, Johannesburg 1710, South Africa; 4 School of Chemistry and Material Science, The Technical University of Kenya, Nairobi P.O. Box 52428-00200, Kenya; 5 Department of Chemistry, School of Physical and Chemical Sciences, Faculty of Natural and Agricultural Sciences, North-West University (Mafikeng Campus), Private Bag X2046, Mmabatho 2735, South Africa

**Keywords:** quantum dots, 17β-estradiol (E2), endocrine-disrupting compounds (EDC), estrogen receptor-alpha (ER-α), hormone, nanomaterials, SnSe

## Abstract

17β-estradiol (E2) is an important natural female hormone that is also classified as an estrogenic endocrine-disrupting compound (e-EDC). It is, however, known to cause more damaging health effects compared to other e-EDCs. Environmental water systems are commonly contaminated with E2 that originates from domestic effluents. The determination of the level of E2 is thus very crucial in both wastewater treatment and in the aspect of environmental pollution management. In this work, an inherent and strong affinity of the estrogen receptor-α (ER-α) for E2 was used as a basis for the development of a biosensor that was highly selective towards E2 determination. A gold disk electrode (AuE) was functionalised with a 3-mercaptopropionic acid-capped tin selenide (SnSe-3MPA) quantum dot to produce a SnSe-3MPA/AuE electroactive sensor platform. The ER-α-based biosensor (ER-α/SnSe-3MPA/AuE) for E2 was produced by the amide chemistry of carboxyl functional groups of SnSe-3MPA quantum dots and the primary amines of ER-α. The ER-α/SnSe-3MPA/AuE receptor-based biosensor exhibited a formal potential (E^0′^) value of 217 ± 12 mV, assigned as the redox potential for monitoring the E2 response using square-wave voltammetry (SWV). The response parameters of the receptor-based biosensor for E2 include a dynamic linear range (DLR) value of 1.0–8.0 nM (R^2^ = 0.99), a limit of detection (LOD) value of 1.69 nM (S/N = 3), and a sensitivity of 0.04 µA/nM. The biosensor exhibited high selectivity for E2 and good recoveries for E2 determination in milk samples.

## 1. Introduction

17β-estradiol (E2) is a biologically significant natural steroidal hormone [1]. It forms part of the female reproductive cycle and functions as the dominant estrogen amongst two other natural estrogens, estrone (E1) and estriol (E3) [2]. The normal E2 levels in human blood are <10 pg/mL in children, 10–20 pg/mL in males, 30–400 pg/mL in premenopausal females and 0–30 pg/mL in postmenopausal females [3]. Concentrations of E2 above these levels in the endocrine system can lead to serious health problems. Estradiol-β contamination is often caused by excreted waste from animals and human beings; it is then distributed into our ecosystem by leaching into domestic and municipal wastewater [4]. The consumption of dairy products such as milk and eggs are other well-known sources of exposure to hormonal estrogens [5,6]. Unlike other estrogens, E2 is not completely and efficiently metabolised in the liver [7]. Hence, its un-metabolised form interferes and triggers discrepancies in the secretion, functioning and production of natural hormones in the endocrine system [3]. Estradiol-β is classified as an endocrine-disrupting compound (EDC) [8]. Exposure to a broad range of EDC has been reported to be caused by uncommon agricultural practices [9] and contaminated domestic and municipal wastewater runoffs [10]. The adverse effects triggered by exposure to elevated E2 concentrations levels have also been linked to population-ratio-imbalances amongst aquatic species [11], menstrual problems [12] and proliferation of cancerous cells [13] in human beings. Estradiol-β can also stimulate inconsistencies during the production of other hormones in the body [14].

The detection of E2 concentration levels is currently achieved through concentration-dependent assays from methodologies such as high-performance liquid chromatography (HPLC) [15], enzyme-linked immunosorbent assay (ELISA) [16] and liquid chromatography coupled to mass spectrometry (LC-MS) [17]. These techniques are time-consuming, usually afford high detection limits, have restrictive experimental conditions, and require highly trained personnel [4]. Thus, the detection of E2 requires improved detection procedures to address the above-mentioned limitations. Electrochemical biosensors have been proven in the literature to be one of the promising techniques that have the potential to be applied for the detection of many types of analytes, including environmental pollutants and biological analytes, due to their high specificity and accuracy, even in complex matrices [1]. Most electrochemical biosensors for E2 use enzymes [1,18], aptamers [19] and antigens/antibodies [20,21] as the bio-receptors on the sensor platform. Immunosensors systems are limited to indirect detection [22], while aptasensors require complex, labour-intensive and time-consuming chemical labelling of the aptamer sequence [23]. The main limitation of enzyme-based biosensors is their ability to catalyse more than one substrate, especially those with chemical structures similar to that of the target analyte [24]. This leads to non-specificity of the biosensor, which could further result in biosensors responding to interferences instead of the target analyte. To address these shortcomings, the sensing platform can be designed such that the biomolecule is a specific affinity receptor to the target analyte. Moreover, enzymes exhibit similar electrocatalytic behaviour in the presence of variable substrates, determined by redox over-potentials ascribed to either oxygen evolution, oxygen reduction or the evolution of peroxides [25]. The oxygen reduction reaction (ORR) and oxygen evolution reaction (OER) redox peaks can also be generated due to the presence of either an intersect of catalysts, oxygen, and hydrogen species in the electrolyte solution [26]. Hence, observing the change in the ORR and OER could result in false-positive electrochemical responses. Bio-catalysis research is now diverting into the use of alternative biomolecules, such as engineered enzymes, in order to improve parameters such as stability and selectivity [27]. Nevertheless, the enzyme modification processes are extremely complex and costly. Subsequently, cell receptors are uniquely activated by specific ligands.

Recent research evidently shows that the successful chemical modification of nanomaterials, particularly quantum dots with biomolecules, invigorates the evolution of new properties that make them applicable during the development of several novel biosensors [28,29], bio-imaging devices [30,31] and for other biological applications. Furthermore, quantum dot nanomaterials exhibit excellent physio-chemical properties, such as a high surface area-to-volume ratio, good stability, and superior electronic properties. This permits them to be applied during the fabrication of electrochemical biosensors and sensing devices [32].

**Figure 1:**
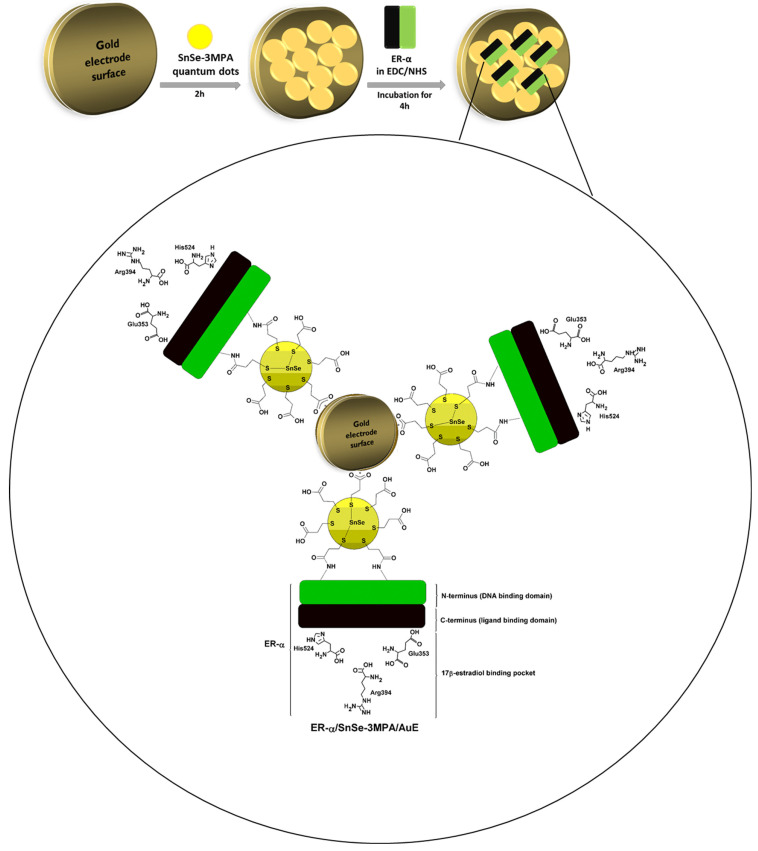
The schematic diagram illustrating the design of the ER-α/SnSe-3MPA/AuE receptor-based biosensor.

In this work, 3-mercaptopropionic acid-capped SnSe quantum dots (SnSe-3MPA) were produced and coupled to an estrogen receptor alpha (ER-α) through an N-hydroxysuccinimide (NHS) and 1-ethyl-3-(3-dimethylaminopropyl) carbodiimide hydrochloride (EDC) covalent-coupling chemistry in order to develop a highly selective electrochemical biosensor for E2. The design strategy of the proposed bio-electrode system is based on the physiological and selective activation of ER-α by E2. The carboxyl functional groups around the surface of the quantum dots ensured effective covalent coupling between the quantum dots and the conserved primary amines from amino-acid functional groups of the cell receptor ER-α. This study demonstrated that the 3-mercaptopropionic acid-capping ligand played an important role in enhancing the susceptibility of ER-α to bind to quantum dots deposited on the gold electrode surface. Moreover, the quantum dots were able to facilitate the effective exchange of electrons and enriched the attachment of the ER-α biomolecule onto the surface of the electrode. The work gives insights into one possible approach to develop cell-surface receptor/nanomaterial-based bio-catalysts as mechanisms for the selective capturing, monitoring, profiling, and inhibition of molecules of a similar nature to E2. These nano-biocatalysts could be employed to suppress molecules of biological relevance or bio-threat, such as contaminants, bacteria, viruses, and other pathogens. The electrochemical receptor-based nano-biosensor was methodically developed to ensure the activity of the cell receptor is well-kept during the electrochemical detection of E2; the outcomes were exceptional. Moreover, the fabricated biosensor demonstrated excellent electrocatalytic activity during the detection of 17β-estradiol and was highly selective. This is attributable to the physiological affinity of the ER-α for the ligand E2. Figure 1 illustrates the design principle of the biosensor. ER-α is integrated on the SnSe-3MPA quantum dots through its N-terminus/DNA binding domain via an EDC/NHS cross-linking reagent. The ligand-binding domain or binding pocket of the ER-α is responsible for selectively capturing the E2 analyte.

## 2. Materials and Methods

### 2.1. Materials

All reagents used in this study were of analytical grade and purchased from Merck (Johannesburg, South Africa). Sodium borohydride of 99% purity grade, 99% pure selenium (Se) group (IV) metalloid powder, 3-mercaptopropionic acid (3MPA), sodium dihydrogen phosphate (NaH_2_PO_4_), disodium hydrogen phosphate (Na_2_HPO_4_), 17β-estradiol (E2), N-hydroxysuccinimide (NHS), and 1-ethyl-3-(3-dimethylaminopropyl) carbodiimide hydrochloride (EDC-2) were all purchased from Merck. The tin chloride dihydrate (SnCl_2_.2H_2_O) of 99% purity grade used as a metal precursor for the quantum dots was purchased from Merck. A 50% glycerol solution, ethyldiaminetetraacetic acid (EDTA), a tris-acetate buffer of pH 7.4, and absolute ethanol were purchased from Merck. The 0.1 M phosphate buffer solutions of pH 7.4 were prepared from sodium dihydrogen phosphate and disodium hydrogen phosphate in double deionised water filtered by a Millipore (Merck KGaA, Darmstadt, Germany) filtering system.

### 2.2. Methods

#### 2.2.1. Characterization Techniques

Transmission electron microscopy (TEM) images were recorded on a Tecnai G2 F20X-Twin MAT 200 kV Field Emission Transmission Electron Microscope (Field Electron and Ion (FEI) Company, Hillsboro, OR, USA). The TEM samples were dispersed on a copper-coated TEM grid. Ultraviolet-visible (UV-Vis) absorption measurements of samples were carried out in quartz cuvettes on a Nicolet Evolution 100 UV-Vis Spectrophotometer (Thermo Electron Corporation, Waltham, MA, USA). Atomic force microscopy (AFM) topographic imaging was conducted using a Veeco NanoMan V AFM system (Bruker Corporation, MA, USA) operated on a taping mode. Fluorescence spectrometry measurements were performed with a Nanolog Spectrofluorometer (HORIBA Scientific, Cedex, France). All electrochemical experiments were carried out with a BAS 100 W Electrochemical Analyzer (Bioanalytical Systems Incorporated (BASi), West Lafayette, IN, USA). A 10 mL electrochemical cell with a conventional three-electrode setup was used. A gold disk electrode with a geometric area of 0.0201 cm^2^ was used as the working electrode, while a Ag/AgCl (3 M NaCl) was used as the reference electrode. Both electrodes were purchased from BASi. Platinum wire (Merck, Johannesburg, South Africa) was used as the counter electrode. The working electrode was polished with alumina powders (Buehler, Lake Bluff, IL, USA) of 0.05, 0.3 and 1 μm particle sizes and sonicated in deionised water, followed by methanol. In circumstances where the organic components adsorbed on the gold electrode surface, the traces or matrices of the pre-adsorbed materials were efficiently removed by soaking the working electrode in a mixture of hydrogen peroxide and sulphuric acid (i.e. the piranha solution) for a few minutes.

#### 2.2.2. Preparation of the Tin Selenide Quantum Dots Capped with the 3-Mercaptopropionic Acid

The 3-mercaptopropionic acid-capped tin selenide quantum dots (SnSe-3MPA) were synthesised at 25 °C. In the first reaction flask, 0.021 g of NaBH_4_ was reacted with 0.021 g of Se powder in 200 mL deionised water used as the solvent. The reaction was ameliorated by a constant flow of nitrogen gas (N_2_) into the precursor solution for 1.5 h. In the reaction, selenium was reduced from a zero (0) to a (−2) oxidation state. The reduction of Se to Se^2−^ using sodium borohydride results in the formation of aqueous NaHSe [33], which inserts the Se^2−^ source into the reaction. In a separate flask, the metal source precursor (i.e., Sn^2+^(3-MPA) metal complex) was prepared by dissolving 0.06 g of the Sn(II)Cl_2_.2H_2_O and 139.2 µL of 3-mercaptopropionic acid (3MPA) (capping agent) in 200 mL of ultrapure water. The reaction was allowed to stir at 700 rpm for a few minutes. Then, the pH of the solution was adjusted to 8 using 1 M NaOH. This resulted in the formation of the Sn^2+^(3-MPA)_x_ complex. The nucleation of the SnSe-3MPA quantum dots was initiated by the addition of 200 mL NaHSe solution dropwise onto the Sn^2+^(3-MPA)_x_ metal complex solution under a constant flow of N_2_ gas onto the solution and vigorous stirring at 700 rpm for 30 min. The oxygen-free environment is crucial during the growth of quantum dots to avoid the formation of metal oxides. The nucleation reaction of the 3-mercaptopropionic acid-capped tin selenide quantum dots was then quenched by placing the quantum dot solution at 4 °C for 5 min. The synthesized quantum dots were then transferred into the freezer at −20 °C and remained stable for more than a month.

#### 2.2.3. Immobilisation of the 3-Mercaptopropionic Acid-Capped Tin Selenide Quantum Dots on the Gold Electrode Surface

The gold electrode surface was immersed into a solution containing tin selenide quantum dots capped with 3-mercaptopropionic acid for 2 h, wherein a resultant SnSe-3MPA/AuE modified surface was obtained. The negatively charged carboxylic acid-capping functional groups of the SnSe-3MPA quantum dots electrostatically adhered on the partially positively hyphenated gold electrode surface. The SnSe-3MPA/AuE was then immersed into a solution of a 1:1 molar ratio of 0.1 mM EDC:0.1 mM NHS for 2 h. This was for the purpose of activating the carboxylic acid groups on the 3-mercaptopropionic acid-capping of the SnSe-3MPA/AuE surface.

#### 2.2.4. Preparation of the Biosensor ER-α/SnSe-3MPA/AuE

The estrogen receptor alpha (ER-α) was introduced onto the EDC/NHS-activated SnSe-3MPA/AuE surface by drop digestion of 0.1 µL of 2 µM ER-α stabilized in 20% glycerol, 50 mM Tris-acetate buffer of pH 7.4, and 1 mM EDTA. The amine groups of the amino acids present in the N-terminus of the ER-α were then allowed to cross-link with the carboxylic acid groups of the SnSe-3MPA/AuE surface for 2 h. The ER-α-modified SnSe-3MPA/AuE electrode was then allowed for ingestion and incubation for an additional hour at approximately 4 °C. The prepared receptor based biosensor surfaces (ER-α/SnSe-3MPA/AuE) were evaluated for their electrochemical properties and used to detect 17β-estradiol.

#### 2.2.5. Calibration of the Biosensor

Briefly, a stock concentration of 300 nM 17β-estradiol was prepared using absolute ethanol 2% in a sub-zero cold 0.1 M phosphate buffer solution of pH 7.4. For electrochemical measurements, equal volumes of 20 µL aliquots of 300 nM 17β-estradiol were continuously spiked in an electrochemical cell consisting of 3000 µL volume of 0.1 M phosphate buffer solution, pH 7.4 (i.e., to represent a concentration as alluded to in Section 3.5 and Section 3.6). After each spike addition of the analyte, the working electrode was completely lifted from the electrolyte solution. The solution was stirred for at least two minutes at 10 rpm; then, the electrolyte was allowed to reach equilibrium before each square-wave voltammetry measurement was carried out.

#### 2.2.6. Preparation of Milk Samples

Fresh milk was obtained from a local convenience store in Cape Town, South Africa. A sufficient quantity of milk was pre-treated by centrifugation at 7000 rpm for 5 min, with the purpose of removing the elevated fat content that might interfere with the detection of the analyte of interest. The supernatant was removed, and the milk sample was then diluted with 50 % of 0.1 M phosphate buffer solution (pH = 7.4). Three different volumes of 17β-estradiol (E2) were then spiked into pre-treated milk samples to prepare samples containing un-spiked, 2 nM, 4 nM and 6 nM concentrations of the analyte. Square-wave voltammetry was then used to measure and monitor the responses of the biosensor to different concentrations of E2 in real milk samples.

## 3. Results and Discussion

### 3.1. Size and Morphological Analysis of the SnSe-3MPA Quantum Dots

**Figure 2:**
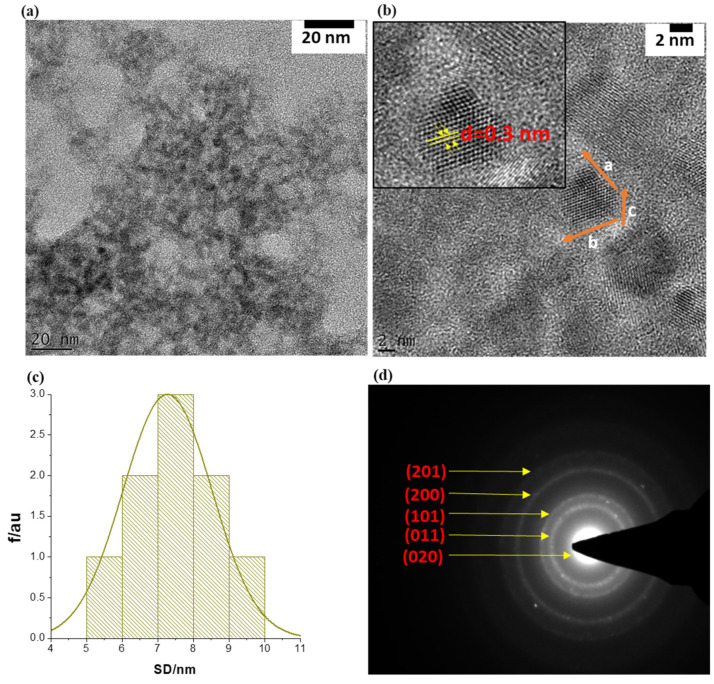
(**a**) The TEM image of the SnSe-3MPA quantum dots at 20 nm resolution scale. (**b**) TEM high-resolution micrograms of single SnSe-3MPA quantum dots (2 nm resolution scale). (**c**) The corresponding particle size distribution histogram (i.e., SD = size diameter), and (**d**) the SAED pattern of single-crystal SnSe-3MPA quantum dots.

Particle sizes, morphology, and the crystallinity of the SnSe-3MPA quantum dots were investigated using transmission electron microscopy (TEM) and selected area electron diffraction (SAED) analysis, the findings of which are shown in Figure 2a–d, respectively. The TEM micrographs depicted in Figure 2a (resolution scale = 20 nm) and Figure 2b (resolution scale = 2 nm) revealed that the particles formed were of near-spherical shape, poly-crystalline, and possessed particle diameters of less than 10 nm. The average particle size (APS) diameter of the prepared SnSe-3-MPA quantum dots was estimated using the particle size distribution histogram, shown in Figure 2c. The particle size distribution curve (Figure 2c) revealed that the SnSe-3MPA quantum dots had average particle diameters of 7.5 ± 1.5 nm.

**Figure 3:**
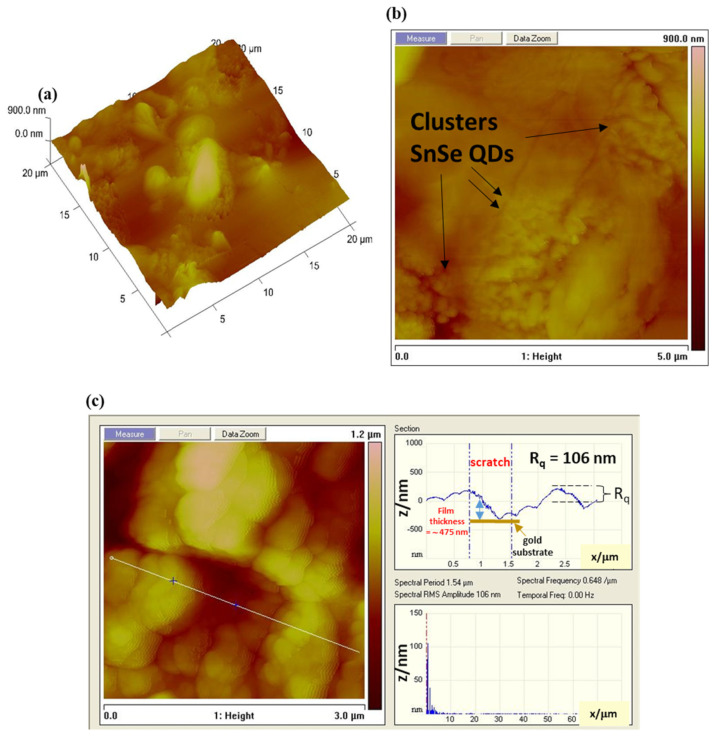
(**a**,**b**) The atomic force microscopy (AFM) and topographic mapping of the SnSe-3MPA quantum dot nanocrystals deposited on a silicon substrate. (**c**) The cross-sectional AFM image and the surface roughness measurements.

The crystallographic pattern of the SnSe-3MPA quantum dots was resolved through lattice fringe mapping using a single quantum dot crystallite, as highlighted in the HRTEM image shown in Figure 2b. The quantum dot lattice fringe simulations of the real space vector lengths, a, b, and c, where; a ≠ b ≠ c, confirmed the formation of orthorhombic SnSe nanocrystal structures. The lattice fringe simulations also allowed for computing the d-inter-planar spacing, where d = 0.3 nm (i.e., d = ~3 Å) was obtained. The observed d-atomic spacing was consistent with SnSe quantum dots produced using the electrochemical exfoliation method reported by Jin Li and co-workers, which they indexed to the (011) lattice plane of SnSe [34]. The crystal structure/configuration of the SnSe-3MPA quantum dots was further examined by means of the SAED pattern shown in Figure 3d. The SAED pattern revealed orthogonally oriented bright spots that could be indexed to the superb crystalline nature of the SnSe quantum dots [35]. Furthermore, the d-inter-planar distances from the presented SAED pattern could be indexed to (201), (200), (101), (011) and (020) of the orthorhombic SnSe crystal phase. The d-inter-planar spacing was converted into crystal plane representations or Miller indices using the crystallographic reference database of orthorhombic SnSe crystal structures reported by Gourab Karmakar and co-workers [36].

### 3.2. Surface Topography Analysis of the SnSe-3MPA Quantum Dots

The surface topography features of 3-mercaptopropionic acid-capped SnSe quantum dot films were further investigated using atomic force microscopy (AFM). The AFM micrographs in Figure 3a–c demonstrate well-organised quantum dots with obvious surface defects. The aggregated clusters of SnSe-3MPA quantum dot crystallites were well visible in the AFM topographic images displayed in Figure 3a,b. Quantum dot-derived films are known to exist as poly-crystallites with high degree of aggregation [37]. The surface roughness analysis of the SnSe-3MPA quantum dot films was calculated from the power spectral density (PSD) of the AFM line scan, shown in Figure 3c. The PSD analysis revealed that the SnSe-3MPA quantum dot films had a root-mean-square (RMS) height (Rq) of 106 nm. The obtained surface roughness was 100 magnitudes higher than that obtained in literature by H. Choi and co-workers [38] when studying the topography characteristics of supersonic spray-coated PbS quantum dots. The average surface roughness of materials or modified surfaces is related to their surface adhesion capabilities, implying that rough surfaces demonstrate better adhesion in comparison to smooth surfaces. The surface roughness characteristics of the SnSe-3MPA quantum dot would strongly facilitate the effective attachment of the estrogen-receptor alpha biomolecule. Surface roughness can alternatively be represented by means of the root-mean-square (RMS) height, α, through the given Equation (1) [39].

α = √{E[h^2^(x)]s − {E[h(x)]_s_}^2^ (1)

The representation E describes the probable value of a random inconstant; h represents the height of the surface. x is the Cartesian plane position. Zhang and co-workers [39] studied the mechanistic interaction of electrons with random rough surfaces and revealed that the irradiation of electrons in solid surfaces (i.e. described as a secondary electron (SE)) is highly dependent on the surface roughness. In their simulation of various models, they established that rough surfaces significantly increase the co-relation length (l) to surface roughness height α ratio, measured at a fixed energy and electron incident angle. Implying that smaller lc/α reduces the probability of electrons to diffuse in a solid metal support [39]. Hence, slightly rough surfaces would predominantly facilitate the effective scattering and diffusion of electrons. The electrochemical reactions are uniquely driven by the exchange and scattering of electrons and ions at the interface of the electrode. Hence, rough nano-film interfaces would be preferred, attributable to their foreseeable ability to facilitate the effective transfer of electrons. Hence, rough surfaces would most likely exhibit improved redox reactivity. The film thickness was estimated by computing the indention depth height of the SnSe-3MPA quantum dots film deposited on a silicon substrate. Alternately, a scratch could be created on a modified surface using the AFM tip, where the same scratch depth represents the accurate measure of the film thickness. The indentations on the interface of the SnSe-3MPA quantum dots film as it appears in Figure 3c, represent the exposed substrate sites and could also be used to estimate the film thickness. In this case, the film thickness was measured in the −z Cartesian plane and estimated to be ~475 nm. Very thin films are characterised by the improved rate of diffusion and accelerated transfer of electrons, in addition are less prone to fouling. Michel Fromm utilised the AFM fitted with an aluminium-coated silicon nitride tip operated at the tapping/contact mode resonance frequency of 300 kHz to scratch the DNA surface [40]. The authors were able to estimate the thickness of the DNA layer from the scratch depth analysis.

### 3.3. Electrochemical Properties of the SnSe-3MPA Quantum Dots

**Figure 4:**
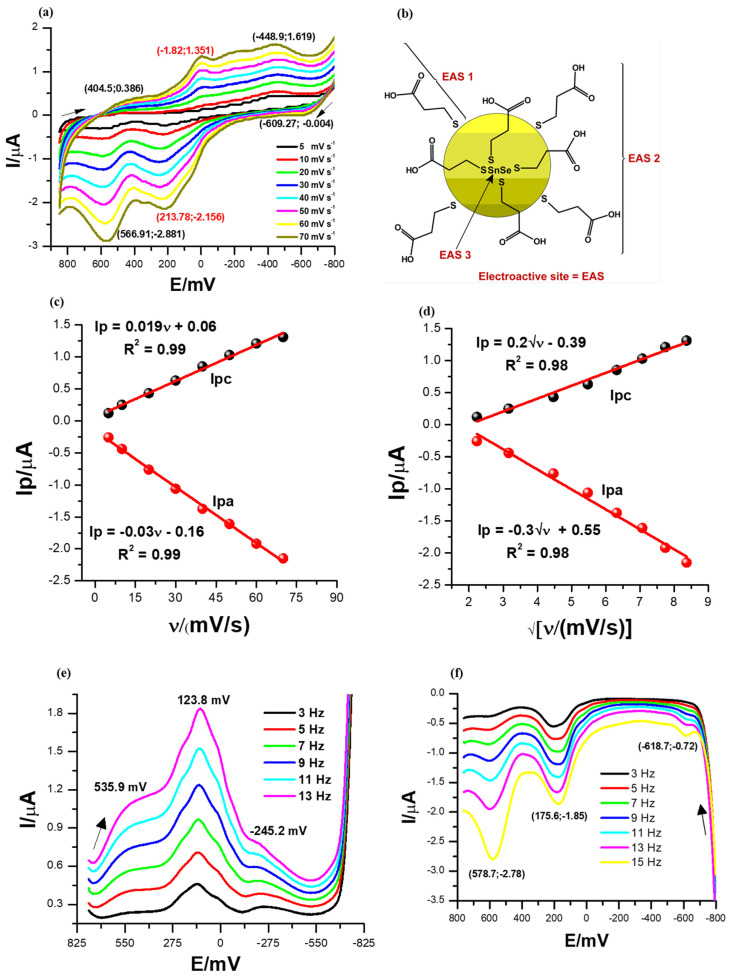
(**a**) Cyclic voltammetry (CV) scan. (**b**) The electroactive sites present in the SnSe-3MPA quantum dots. The arrows indicate the direction of the scan. (**c**) Linear plots of the peak current versus scan rate. (**d**) The cathodic and anodic redox peak currents versus square root of scan rate plots for the SnSe-3MPA quantum dot-modified electrode interface derived from CV measurements. (**e**,**f**) Square-wave voltammetry (SWV) responses of the SnSe-3MPA quantum dot films deposited on a gold electrode surface in 0.1 M phosphate buffer solution of pH 7.4 at varying frequencies from 3 to 13 Hz (i.e., at the fixed square-wave amplitude = 25 mV and step size= 2 mV).

The cyclic voltammograms of the SnSe-3MPA quantum dots were recorded at a potential window between 800 and −800 mV and a scan rate range between 5 and 70 mV/s in a 0.1 M phosphate buffer solution of pH 7.4 at 25 °C. The cyclic voltammograms shown in Figure 4a and square-wave voltammograms in Figure 4e,f represent 2 µL of the SnSe-3MPA quantum dot films deposited on a gold disk electrode surface. The cyclic voltammograms depicted in Figure 4a exhibited distinct reduction peaks at Epc ≈ 404.5 mV, −1.82.0 mV, and −444.23 mV and oxidation peaks at Epa≈ −609.3 mV, 213.78 mV, and 566.91 mV. These peaks are related to the redox transformation of the electroactive triad-sites present in the SnSe-3MPA quantum dots, as illustrated in Figure 4b. The electroactive fragments responsible for the overall electrochemical behaviour of SnSe-3MPA quantum dots are also shown in the Supplementary Document and represented by Figure S5 in Supplementary Material. The peak-to-peak separation values (ΔEp) obtained from cyclic voltammetry for all redox processes involved were >57 mV, indicating rather more complex and coupled multi-electron transfer reactions occurring at the confined electrode interface [41]. The redox reaction with the peak separation ΔE_p_ ~215.6 mV centred in the cyclic voltammograms shown in Figure 4a was suggested to be the redox transformation reaction occurring at the Fermi or valence electronic band [42] of the SnSe-3MPA quantum dots. Cathodic and anodic square-wave scans/voltammograms presented by Figure 4e,f each revealed a set of 3 redox peaks at formal potentials of {534 mV, 123.8 mV, and −245 mV} and {578.7 mV, 175 mV, and −618.7 mV}, respectively. These redox peaks were consistent with the redox peaks obtained from cyclic voltammetry. For semiconductors, the indirect and direct band gaps are created by a combination of electron-hole pair systems with an abundance of free electrons tunnelling within these energy bands. This simply suggests that electrons freely oscillate between the electronic levels in the effect of the applied potential or energy. Hence, the redox activity of materials is strongly dependent on the availability of oscillating free electrons. In this study, the high redox activity displayed by the quantum dots at variable redox potentials was attributed to the tunnelling of free electrons and the good distribution of defects and hole sites within the SnSe-3MPA quantum dots.

The splitting effect of the electrochemical signal at the redox peak centred at Epc = −1.82 mV and Epa = 213.78 mV, as highlighted in the CV profiles in Figure 4a, might also be a good indication of a multi-electron transfer reaction that occurred at the modified electrode surface. The electrochemical interfacial properties of the SnSe-3MPA quantum dot films were further investigated by means of the electrochemical surface adsorption kinetics presented by the linear plots of Figure 4c of peak currents vs. scan rates from cyclic voltammetry analysis. The adsorption kinetics relate the electron transfer rates with the redox peak currents, Ip(s). The peak current (Ip) versus scan rate (ν) plots resulted in linear profiles with the equations Ip = 0.019ν + 0.06, R^2^ = 0.99 and Ip = −0.03ν − 0.16, R^2^ = 0.99 for the reduction and oxidation processes, respectively (i.e., at Epc = −1.82 mV and Epa = 213.78 mV). This confirmed that both cathodic and anodic reactions occurring at the electrode interface occurred through the adsorbed SnSe-3MPA quantum dot species modified at the interface of the electrode. The surface concentrations (Γ)s of the SnSe-3MPA quantum dots deposited on the gold electrode surface were calculated using Equation (2) [41].

Ip = |n^2^F^2^AΓν|/4RT (2)

where the symbol Ip_,_ represents the peak current, n = 2 is the number of electrons transferred in a redox reaction for any given reversible and quasi-reversible electrochemical system, F is the Faraday constant, A is the geometric area of an electroactive substrate, R is the universal gas constant, and T represents the absolute temperature in Kelvins. The number of electrons (n) was estimated using the expression E_p/2_ = E_1/2_ ± (29 mV/n) [43], where E_1/2_ is the half-wave potential (i.e., E_1/2_ ~E^0^′) and E_p/2_ is the mid-peak potential between Epa and Epc, which are anodic and cathodic peak potentials, respectively. Surface concentrations of 8.075 × 10^−13^ mol/cm^2^ and 1.275 × 10^−12^ mol/cm^2^ were computed for the cathodic and anodic processes, respectively. This was an indication that very thin films of SnSe-3MPA quantum dots adsorbed on the electrode surface were capable of exchanging electrons or ions with the electrolyte solution. The electrochemical properties of the SnSe-3MPA quantum dots were further interpreted using the Randles–Ševčík kinetics demonstrated by Equation (3) [44], utilising the same redox peaks. The Randles–Ševčík plots in Figure 4d were linear for both the cathodic and anodic processes and resulted in the equations Ip = 0.2√ν − 0.39, R^2^ = 0.98 and Ip= −0.3√ν + 0.55, R^2^ = 0.98, respectively. The linearity of the Randles–Ševčík kinetic plots confirmed that the redox reactions occurring at the SnSe-3MPA quantum dot-modified gold electrode were also diffusion-controlled. Hence, this confirmed that the observed redox kinetics occurring at the SnSe-3MPA-modified gold electrode interface were controlled by both adsorption and diffusion processes. The diffusion coefficient(s) were calculated using Equation (3).

Ip = 0.4463 nFAC*√[nFDν/RT](3)

The symbols Ip, n, F, A, R and T have been described previously under Equation (2). The parameters C* and D denote the concentration of the bulk of the electrolyte solution and the diffusion coefficient, respectively. The anodic and cathodic processes of the surface-bound SnSe-3MPA quantum dots were characterized by diffusion coefficient(s) (D) of 3.38 × 10^−11^ cm^2^/s and 5.07 × 10^−11^ cm^2^/s. This was an indication that the anodic and the cathodic processes under evaluation occurred at similar rates and possibly involved the same intermediates.

Furthermore, the dynamic kinetic properties of thin films of SnSe-3MPA quantum dots were demonstrated by the Stokes–Einstein–Sutherland estimation expression. The equation relates the kinetic diffusion characteristics of the quantum dot films with the dynamic viscosity η, as presented in Equation (4) [45], where K_B_ is Boltzmann’s constant, (T) is the absolute temperature, (r_solute_) is the radius of the solute, and D is the diffusion coefficient magnitude of the SnSe-3MPA quantum dot films.

η* = K_B_T/6πr_solute_D (4)

Using Equation (4)**,** the dynamic viscosity of the electroactive SnSe-3MPA quantum dot-modified films for both the cathodic and anodic processes were calculated to be 8.67 × 10^−13^ kg/ms and 5.7 × 10^−13^ kg/ms, respectively. The low value of the dynamic viscosity can be ascribed to effective tunneling and migration of the electroactive species within the SnSe-3MPA quantum dot-mediating layer. The SnSe-3MPA quantum dot-modified gold electrode surfaces exhibited excellent redox behaviour for their intended application as the mediating material during the fabrication of the proposed receptor-based biosensor.

### 3.4. Optimisation of the Electroanalytical Signal of the ER-α/SnSe-3MPA/AuE Biosensor during the Detection of 17β-Estradiol

**Figure 5:**
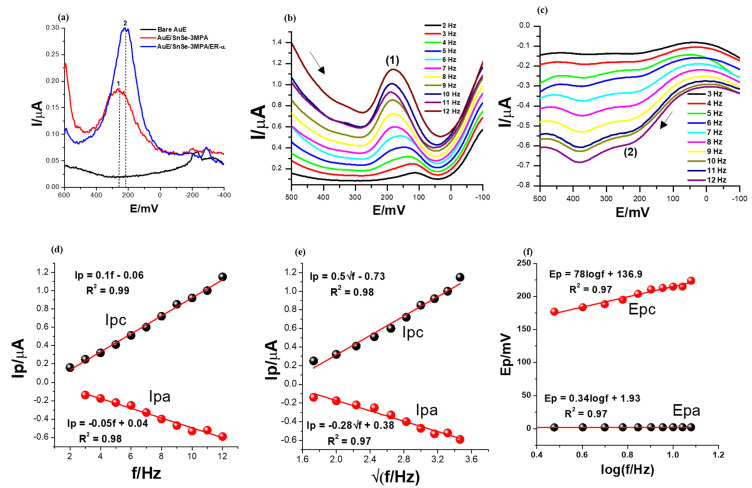
The SWV responses of (**a**) bare, SnSe-3MPA- and ER-α/SnSe-3MPA/AuE-modified electrodes in the presence of 50 nM 17β-estradiol in 0.1 M phosphate buffer solution of pH = 7.4, recorded at a frequency of 15 Hz, Estep = 2 mV, and Esw = 25 mV. The arrows indicate the direction of the scan. (**b**,**c**) Forward and reverse SWV responses of the ER-α/SnSe-3MPA/AuE biosensor in the electrolyte solution containing 50 nM 17β-estradiol in 0.1 M phosphate buffer solution of pH = 7.4 at varying frequencies between 2 and 12 Hz (Esw = 25 mV and Estep = 2 mV were kept constant). (**d**) Peak current vs. frequency, (**e**) peak current vs. square root of frequency, and (**f**) peak potentials vs. log (frequency) plots.

The electrochemical characteristics of the constructed biosensor were first examined using different modified electrodes: bare AuE, SnSe-3MPA/AuE and ER-α/SnSe-3MPA/AuE. The square-wave voltammetry technique is the most powerful and most preferred method for evaluating mostly interfacial redox reactions due to its capability to eliminate redox responses generated from non-Faradaic/charging currents by simultaneously measuring the total current at ΔEp steps [46,47]. Square-wave voltammetry is widely employed for studying complex and multiple redox reactions occurring in biomolecules [46] and complex catalytic electrochemical processes [48]. Square-wave voltammograms demonstrating the electrochemical behaviours of different electrodes in the presence of 50 nM 17β-estradiol in 0.1 M phosphate buffer electrolyte solution of pH 7.4 are displayed in Figure 5a. A square-wave frequency (f) of 15 Hz, an amplitude (Esw) of 25 mV, and a step potential (Estep) of 2 mV (translated to a scan rate (ν) of 30 mV/s by the relation of ν = f/ΔE [49]) were used. The ER-α/SnSe-3MPA/AuE biosensor resulted in significantly large square-wave response peak currents, followed by the SnSe-3MPA/AuE-modified surface, evaluated under the same conditions. The bare gold electrode did not result in any redox peak at the potential window of interest. Conversely, the SnSe-3MPA quantum dot-modified gold electrode surface resulted in a slightly pronounced redox peak observed at Ep^0′^ = 252 mV. A slight cathodic shift in the peak potential was observed upon conjugation of the SnSe-3MPA quantum dots with the ER-α bio-receptor to a formal potential, Ep^0′^, of 216 mV. Ascribed to their ultra-fine size, quantum dots could be efficiently employed to enhance the surface area-to-volume ratio (which, in this case, translates to surface electrochemical reactivity) or the sensitivity of the many electrode surfaces. Similarly, they could be employed as blocking agents to impede unspecific binding. Bio-receptors are often used as molecules to facilitate and enhance the selectivity of bio-sensing electrodes, and in this circumstance, ER-α was employed to selectively target the 17β-estradiol analyte. Another interesting elucidation was that both SnSe-3MPA quantum dots and ER-α demonstrated redox behaviour at analogous redox potentials, despite the fact that the biosensor exhibited a much more improved electrochemical signal. Hence, the SnSe-3MPA quantum dots were the best candidates for mediating the electron transfer reaction occurring at the interface of this reported bio-sensing system.

In order to understand the broad electrochemical and redox kinetic behaviour of the ER-α/SnSe-3MPA/AuE biosensor film, the square-wave voltammograms were recorded at varying square-wave frequencies from 2 to 12 Hz for both the cathodic and anodic scans under the same electrolyte conditions of the phosphate buffer solution containing 50 nM 17β-estradiol analyte. The square-wave kinetic studies also allowed for choosing the optimum experimental conditions that resulted in well-defined square-wave voltammetry signals. The step potential (Estep) and the square-wave amplitude (Esw) were fixed at 2 mV and 25 mV, respectively.

As depicted in the cathodic and anodic square-wave voltammograms of Figure 5b,c, at (Ep^0^′) = 159 ± 28.43 mV and 202.43 ± 15.67 mV (labelled (1) and (2), respectively), the square-wave response peak currents increased systematically with increasing frequencies from 2 to 12 Hz. However, for both forward and reverse scans, this was accompanied by a corresponding slight anodic peak potential shift observed with increasing frequency. This can also be observed in the plots of peak potential vs. the log (frequency) presented in Figure 5f. This could be attributable to the sluggish nature of the redox process occurring at the interface of the biosensor electrode.

The first criterion for diagnosing the reversibility and the nature of the electrode reaction using square-wave voltammetry is by means of studying the dependence of the net square-wave peak currents with the square root of the frequency (f) [47,49,50]. The plots of peak currents for the forward and reverse scans vs. the square root of frequencies shown in Figure 5e gave rise to linear profiles with the equations Ipc = 0.5√f − 0.73 and the regression coefficient (R^2^) =0.98 and Ipa = −0.28√f + 0.38, R^2^ = 0.98 confirming good linearity. The parameters Esw = 25 mV and Estep = 2 mV were kept constant. Subsequently, for a completely reversible system, the formal potential (i.e., peak potential) must be independent of both the frequency and square-wave amplitude [47]. In this scenario, the relationship between the formal potential and the frequency was studied (as shown in Figure 5d); a linear profile indicated that the two parameters were dependent on each other. Hence, it could be concluded that the redox reaction occurring at the electrode interface was not completely reversible. Theoretical data suggest that for surface-confined reactions occurring at the electrode interface, the dimensionless current (Ψ) generated from the square-wave voltammetry scan can be expressed as either Ψ = I/nFAC*√Df [51,52] or Ψ = (I/nFAΓf) [49,51] for the diffusion and adsorption-controlled electrode kinetics, respectively. The linear relationship between generated square-wave currents with varying frequencies (Figure 5d) could be attributed to the adsorption-controlled redox kinetics. Consequently, the surface-confined reaction dominant at the electrode interface could also be diagnosed as diffusion controlled and ascribed to the linear dependence of the formal potentials with the √f (Figure 5e). These findings further confirmed that the analyte was dispersed in a susceptible environment that allowed for an efficient diffusion process and the mass transport of the electroactive species between the bulk of the electrolyte and the electrode interface. As evidenced by Figure 5b,c, higher square-wave frequencies resulted in well-defined and high-amplitude characteristic redox peaks. Attributable to these prospects, the higher frequencies and forward scans were selected in subsequent studies and for the analytical calibration of the biosensor.

### 3.5. The Detection of Different Concentrations of 17β-Estradiol Using the Receptor Sensor ER-α/SnSe-3MPA/AuE (Anaerobic Conditions)

**Figure 6:**
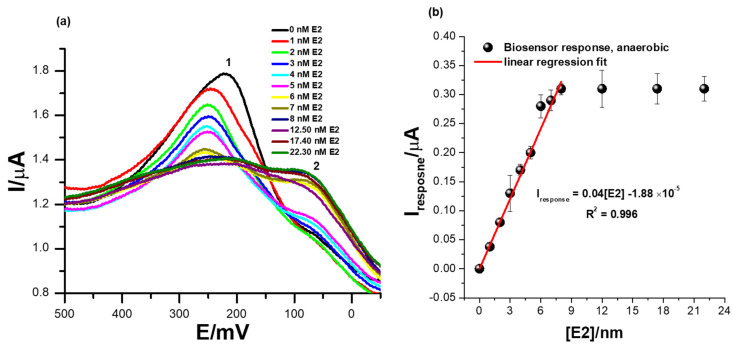
(**a**) Square-wave voltammetry responses of the ER-α/SnSe-3MPA/AuE bio-sensing films at successive additions of 17β-estradiol (i.e., 1–22.3 nM) in 0.1 M phosphate buffer solution of pH 7.4 in the absence of oxygen molecules (anaerobic conditions) and (**b**) the corresponding calibration plot (square-wave voltammetry frequency of 15 Hz, Estep = 2 mV, and Esw amplitude = 25 mV).

Figure 6a shows the square-wave voltammetry responses of the biosensor at successive additions of 17β-estradiol in 0.1 M phosphate buffer solution of pH 7.4 at a square-wave voltammetry frequency of 15 Hz (step size = 2 mV and amplitude = 25 mV) and in the absence of dissolved oxygen species. The ER-α/SnSe-3MPA/AuE biosensor exhibited well-pronounced redox peaks at Ep^0′^ = 217 ± 12 mV at a zero concentration of 17β-estradiol. Increasing the concentration of 17β-estradiol analyte corresponded to a consistent decline of the peak heights, as seen at the redox peak labelled (2) (Ep^0′^ = 217 ± 12 mV) in Figure 6a. This indicates an innate binding affinity between the 17β-estradiol analyte and the ER-α bio-receptor modified on the biosensor surface. The decreasing square-wave redox peak heights with increasing 17β-estradiol concentration could be attributable to the concentration-dependent and effective binding of the analyte to the active center or active site of the ER-α bio-receptor, which, as a result, induced the inhibition of the redox process occurring at (1). Subsequently, this could be an indication of the formation of the ER-α/17β-estradiol complex, which has an insulating effect on the redox center of the developed cell-receptor-based biosensor. The intermediate product formation was also supported by inconsistent responses observed at the redox peak, labelled peak (2) in Figure 6a, that became prominent upon the addition of higher E2 concentrations. The biosensor reached saturation at E2 concentrations slightly above 8 nM, measured at responses observed at Ep^0′^ = 217 ± 12 mV (i.e., peak (1), Figure 6a. After this concentration, no changes in the SWV redox peak heights were observed; this indicated typical receptor/ligand binding kinetics that are expected to reach saturation and typically fit well or commonly conform to hyperbolic curves. The calibration plot represented by Figure 6b exhibited linear responses of the ER-α/SnSe-3MPA/AuE biosensor over a dynamic linear range from 1 to 8 nM of 17β-estradiol. The regression equation for the biosensor responses toward 17β-estradiol is defined as Iresponse = 0.04 [E2] − 1.88 × 10^−5^, with a correlation coefficient (R^2^) of 0.99, evidencing the outstanding and effective electro-catalysis of the E2 analyte. A limit of detection (LOD) of 1.69 nM (S/N = 3) was obtained. The calculated LOD was drastically lower compared to the limits of detection reported in the literature; this includes assays reported by Bilal Yilmaz and YucelKadioglu using UV-Vis and high-performance liquid chromatography methods, where they obtained detection limits of 555 µM and 36.6 µM, respectively [15].

Other electrochemical biosensors and sensors reported in the literature also exhibited fairly low detections limits. Jinchun Song and co-workers modified a glassy carbon electrode surface with a poly(L-serine) thin film to quantify trace levels of 17β-estradiol; they obtained a LOD of 20 mM [53]. Anqing Wanga and co-workers developed an enzyme-based biosensor using electro-polymerized lysine modified with citric acid graphene and laccase enzyme for 17β-estradiol, where they obtained a limit of detection of 1 × 10^−13^ M [1]. Nevertheless, most enzymes have been reported to catalyze more than one type of substrate; thus, for complex sample matrices, enzymes on the biosensor surface could electro-catalyze alternative substrates rather than the target analytes, resulting in false positives. The fabricated biosensor was highly sensitive towards the electro-catalysis of 17β-estradiol, where a sensitivity of magnitude 0.04 µA/nM was obtained. The obtained LOD of 1.69 nM was lower than the World Health Organisation (WHO)-regulated limit for the allowed concentrations of E2 in drinking water.

### 3.6. The Detection of Different Concentrations of 17β-Estradiol Using the ER-α/SnSe-3MPA/AuE Biosensor Platform in the Presence of Oxygen Molecules

**Figure 7:**
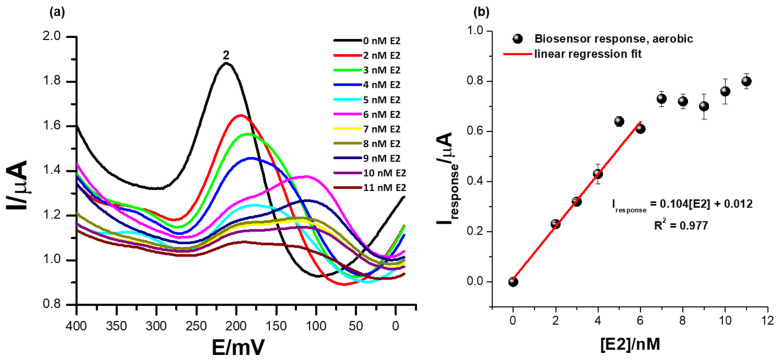
(**a**) The square-wave responses of the ER-α/SnSe-3MPA/AuE biosensor corresponding to different concentrations of 17β-estradiol and (**b**) the corresponding calibration plot of the biosensor in the presence of oxygen molecules (with aerobic conditions and a square-wave voltammetry frequency of 15 Hz, Estep = 2 mV, and Esw amplitude = 25 mV).

Figure 7a illustrates square-wave voltammetry responses illustrating the effect of oxygen during the electrochemical determination of the 17β-estradiol analyte using the ER-α/SnSe-3MPA/AuE biosensor platform. The electrochemical parameters were consistent as in Section 3.5. Concentrations of 17β-estradiol were varied from 1 to 11 nM. Conceding the fact that ER-α preferably binds to the 17β-estradiol compound, proteins [54], DNA molecules [55], and cells are also prone to oxygen poisoning, resulting in the generation of unfavourable reactive oxidative species (ROS) [56]. The formation of ROS is induced by the presence of molecular oxygen and high-energy electrons [54]. ROS exist in the form of O_2_^•-^, ^•^OH, peroxide, super-oxides, etc. [54]. Moreover, ROS can facilitate the loss of biomolecule electrocatalytic activity during the analysis of complex environmental samples where dissolved oxygen molecules are present at pertinent high concentrations. Equation (5) shows the mechanism describing the formation of the biomolecule/radioactive oxygen complex adopted from literature and reported by Hazel J. Shields and co-workers [56].

2O_2_ + 4e^−^ + 4H^+^ + R ⮕٭^−^OR + H_2_O + O_2_^−^ (5)

In the chemical reaction presented by Equation (5)**,** R is any biomolecule with liable active sites for oxygen binding. At a broad potential window, in the presence of oxygen molecules; the ER-α/SnSe-3MPA/AuE biosensor exhibited distinct redox peaks at various formal potentials (as reported in Figure S3 of the Supplementary Document attached). Successive increases in the 17β-estradiol concentrations exclusively influenced distinguishable electrocatalytic activity at a distinct formal potential (Ep^0′^) of 212 mV, peak (2), in Figure 7b. Increasing concentrations of E2 also resulted in a significant reduction in the redox peak heights at (2) and slight shifts of the redox potential. The peak potential at which the consistent responses to E2 concentration increases were observed at peak (2) was consistent with the catalytic redox peak exhibited by the biosensor under anaerobic conditions. However, in the presence of oxygen molecules in the electrolyte and at significantly higher concentrations of E2 from 6 mM and above, secondary coupled redox reactions occurred, assigned to redox peaks observed at Ep^0′^ = 110 mV and shown in Figure 7a. This phenomenon could be a further confirmation that perhaps an intermediate ER-α/E2 complex or an intermediate redox product formed at the interface of the biosensor surface, which subsequently had an affinity for another secondary binding site of the receptor.

The reactive oxygen molecules had little to no influence on the electrocatalytic behaviour and electrocatalytic performance of the fabricated receptor-based biosensor. The biosensor responses under aerobic conditions demonstrated a limit of detection (LOD) of 1.9 nM. The electrochemical behaviour of the biosensor under an oxygen-saturated environment confirmed its potential practicality for real-time measurements. Table 1 illustrates the comparison of similar biosensors reported in literature for the determination of 17β-estradiol.

### 3.7. Selectivity Studies

**Figure 8:**
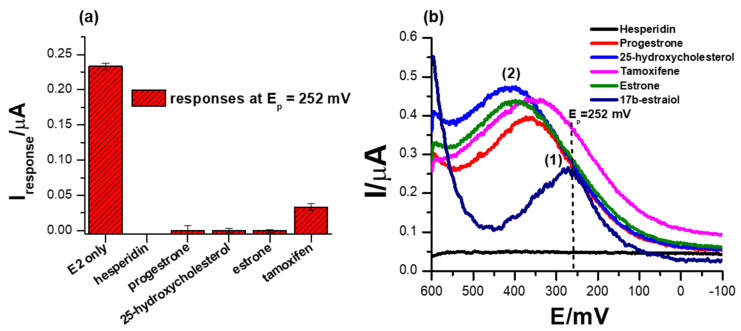
(**a**) The comparison of biosensor responses towards different possible interfering species, 1 µM hesperidin, 1 µM progesterone, 1 µM 25-hydroxycholesterol and 1 µM estrone. (**b**) The SWV responses of the biosensor towards interfering species in 0.1 M phosphate buffer solution, pH 7.4 (square-wave voltammetry frequency of 15 Hz, Estep = 2 mV and Esw amplitude = 25 mV).

Another important parameter to consider when designing biosensors is their selectivity, which is the measure of the ability of the system to only induce an electrochemical signal in response to the analyte of interest. A comparison of different ERα/SnSe-3MPA/AuE biosensor responses to possibly interfering species, including hesperidin, progesterone, 25-hydroxycholesterol, estrone and tamoxifen, are illustrated in Figure 8a,b. These species were chosen on the basis that they are compounds that are catabolized or metabolised in a similar pathway as 17β-estradiol and could possibly induce the activation of ER-α attached to the surface of the biosensor. Moreover, some possess similar structural features to 17β-estradiol. As anticipated, 1 µM of the interfering species hesperidin, progesterone, 25-hydroxycholesterol, and estrone did not induce any SWV responses at the formal potential at which the 17β-estradiol was detected (i.e., Ep^0′^ ~252 mV) as depicted in Figure 8b. This is attributable to the principle that the ER-α mobilised on the electrode interface is solely activated by the analyte of interest, 17β-estradiol (E2). The ER-α/E2 interactions are distinctly observed at the specified redox peak potentials, which are assumed to be the simulation of the active site of the estrogen receptor. The results strongly demonstrate that the designed biosensor for 17β-estradiol was remarkably selective towards the analyte of interest, as proposed earlier. As inspected carefully, a positive response was obtained in the case of tamoxifen, where the presence of 1 µM concentrations of tamoxifen in the electrolyte induced significant amplification of the electrochemical peak current response at the formal potential (E_p_^0′^) of 334.5 mV. This was different to the responses obtained at high concentrations of 17β-estradiol, where a significant drop in the peak potential was observed with increasing concentrations of 17β-estradiol. Tamoxifen is a breast cancer drug that is fabricated to block or inhibit the active site of the nuclear receptor ER-α and is designed to compete with 17β-estradiol for the active site binding [63]. However, upon interaction of tamoxifen with the developed ER-α/SnSe-3MPA/AuE electrochemical system, an enhanced electrochemical signal was obtained. This is also consistent with the responses of the biosensor towards 17β-estradiol, where physiological activation was translated to the inhibition of the ER-α deposited on the electrode surface.

### 3.8. Detection of 17β-Estradiol in Dairy Milk Samples

**Figure 9:**
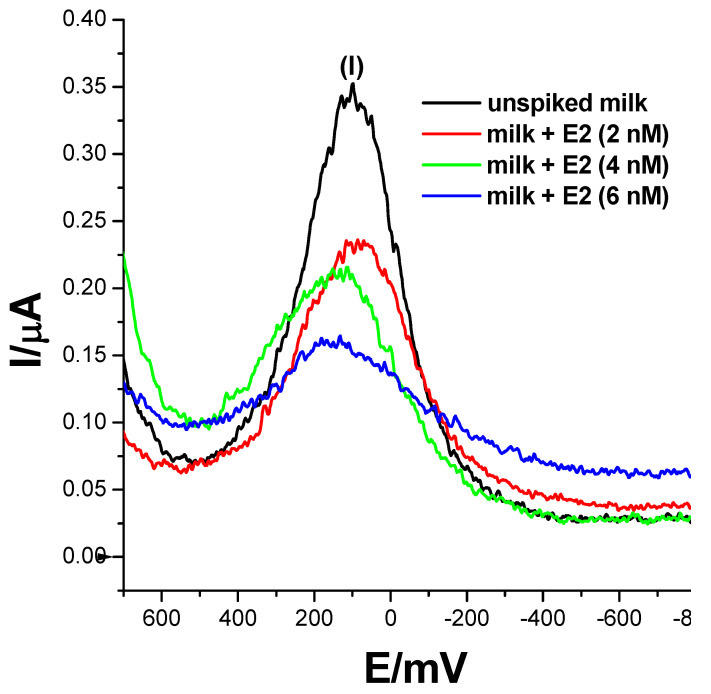
The square-wave responses of the ER-α/SnSe-3MPA/AuE biosensor corresponding to the electrochemical detection of 17β-estradiol (E2) in un-spiked and spiked milk samples; samples were spiked with 17β-estradiol resulting in 2 nM, 4 nM and 6 nM total concentrations in the mixture of 0.1 M phosphate buffer solution and milk (square-wave voltammetry frequency of 15 Hz, Estep = 2 mV and Esw amplitude = 25 mV).

17β-estradiol has been found to be present in momentous concentrations in many dairy products, such as milk, cheese, eggs etc. [5,6]. This is attributable to the use of the E2 compound and its derivatives as a feed additive to boost reproduction in animals and enhance their rate of fertility. 17β-estradiol could also potentially leach into wastewater effluents from agricultural waste, causing estrogen contamination. To assess the practical applicability of the developed biosensor, an analysis of 17β-estradiol was carried-out on raw and spiked milk samples. Three concentrations of 2 nM, 4 nM and 6 nM of 17β-estradiol-spiked samples were prepared using the standard addition method, where 50% of 0.1 M phosphate buffer solution, pH 7.4, was utilised as the diluent. Square-wave responses were then recorded and are displayed in Figure 9. The biosensor did not record any electrochemical changes in un-spiked samples and resembled a voltammogram translating to a 0 nM concentration of E2. However, upon spiking milk samples with 17β-estradiol, the biosensor recorded SWV changes translating to 2.63 nM, 4.45 nM and 5.2 nM, with recoveries of 132.5%, 111.25% and 86.7%, respectively as seen in Table 2. The obtained data strongly suggest that the developed ER-α/SnSe-3MPA/AuE receptor-based biosensor could be used as an accurate and effective method to detect 17β-estradiol in real samples.

### 3.9. The Mechanism of ER-α/SnSe-3MPA/AuE Biosensor toward 17β-Estradiol

ER-α is associated with a 12-helix three-dimensional conformational structure comprised of several distinct domains, such as the C-terminus domain, the DNA-binding domain, the ligand-binding domain, the hinge region, and the N-terminus domain [64]. According to Pimchanok Busayapongchai and co-workers, the ligand binding pocket of the ER-α consists of the amino acids Glu353, Arg394, and His524. These amino acids are responsible for capturing and binding the ligand 17β-estradiol [64].

**Figure 10:**
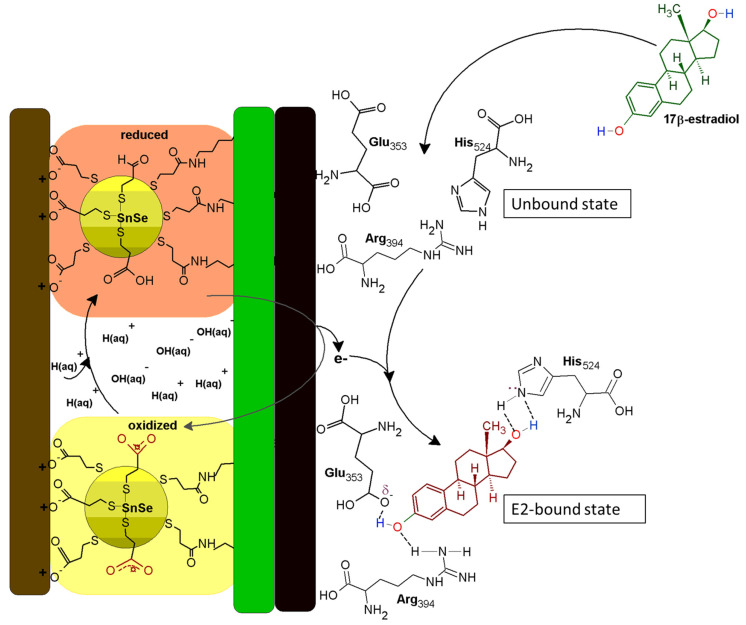
The ER-α/SnSe-3MPA/AuE biosensing electrode mechanism.

The side chain functional groups of Glu353, Arg394 and His524 amino acids effectively interact with the hydroxyl groups of 17β-estradiol at the 3rd and 17th carbon positions, respectively, as in the manner alluded to in Figure 10. This association induces hydrogen bonding and the repositioning and generation of an active activation function 2 (AF2) domain of the receptor. This makes the receptor susceptible to secondary binding with hydrophobic co-activators [64]. The well-defined redox peak at ~217 mV is attributable to the high redox activity demonstrated by the receptor and the exchange of electrons between the ER-α and the SnSe-3MPA quantum dots adsorbed on the gold electrode surface. When 17β-estradiol is introduced to the bulk of the solution, the analyte diffuses and sacks into the binding cavity of the ER-α. Subsequently, the generated and available free electrons hop and are captured by vacant low-lying orbitals of ER-α. This results in a redox electron-transfer reaction and the inhibition of ER-α. The obtained square-wave voltammetry data also ascertained that ER-α becomes electrochemically tainted. The ability of 17β-estradiol to bind to ER-α is dependent on the availability of the binding sites and the position of the analyte proportionate to the receptor sensor interface. The estrogen receptor alpha will preferably induce activation when uniquely bound to 17β-estradiol, making the constructed receptor sensor highly specific and selective towards 17β-estradiol.

## 4. Conclusions

Biomimicry is one of the most efficient well-studied approaches in which natural phenomena are translated into artificial technological systems to resolve modern-age scientific problems. In this work, the same principle of biomimetics was applied to develop a highly selective biosensor for 17β-estradiol. The sensing principle of the developed biosensor is based on the physiological binding and activation mechanism of the nuclear receptor estrogen receptor alpha (ER-α) by the 17β-estradiol compound. The same modus operandi was adopted, translated, and configured into a simple, highly sensitive and selective biosensor for the accurate determination of nano-molar concentrations of an endocrine-disrupting compound, 17β-estradiol. The developed electrochemical receptor-based biosensor detection method demonstrated a greatly improved selectivity and detection limit compared to other methods reported in the literature. Consequently, this was further supported by its high preference for 17β-estradiol compared to possible interference molecules, including hesperidin, progesterone, 25-hydrocholesterol, tamoxifen and estrone. Oxygen poisoning is a common phenomenon observed in many bio-receptor-based in-vitro systems; the developed biosensor showed excellent electro-analytical behaviour under extreme aerobic conditions, hence demonstrating feasibility to be applied in real-world systems. The electrochemical receptor sensor also indicated the capability of recognizing as low as 1.69 nM of 17β-estradiol (its LOD). The limit of detection and the dynamic linear range of the developed electrochemical receptor sensor could, however, be further improved through the exploration of highly conductive hybrid nanomaterials and the use of even higher concentrations of ER-α. The capability of the constructed biosensor to detect E2 in milk samples with acceptable recoveries substantiated its potential to be employed for real-field analysis. The developed biosensors could also be applied for the selective determination of 17β-estradiol in less complex samples, such as treated wastewater, as water demonstrates similar chemical and physical properties as the phosphate buffer electrolyte used during its calibration.

## Figures and Tables

**Table 1:** Comparison of different 17β-estradiol assays.

Biosensor Material	Mode of Detection	Limit of Detection (* = Dynamic Linear Range)	References
Poly(β-CD)/AF1-ADA/ON1/AF2-Au	Electrochemical (DPV)	63.1 fM (*1 × 10^−13^–1 × 10^−9^ M)	[57]
PPY/PMAA-nBA/Aptamer	Electrochemical (DPV)	0.48 pM (*1 × 10^−4^–1 × 10^−12^ M)	[58]
Molecular imprinted polymer/CB	Electrochemical	(*0.10–23.0 × 10^−6^ M)	[59]
SPCE/g-C_3_N_4_/APTES	Electrochemical (DPV)	9.9 × 10^−19^ (*1 × 10^−6^ to 1 × 10^−18^)	[60]
Au@Pt/PEDOT-GO	Electrochemical (DPV)	0.08 × 10^−12^ M (*0.1 × 10^−12^–1 × 10^−9^ M)	[61]
W/MC_0.67_/GCE	Electrochemical (DPV)	8.3 × 10^−9^ (*0.05 × 10^−6^–10 × 10^−6^)	[62]
AuE/SnSe-3MPA QDs/ER-α (receptor)	Electrochemical (SWV)	1.69 × 10^−8^ M (*1–8 × 10^−8^ M)	**This work**

* represents the Dynamic Linear range

**Table 2:** Real sample analysis of 17β-estradiol in dairy milk samples.

Sample	Spiked Concentration (nM)	Detected Concentration (nM)	Recovery (%)	RSD (%, n = 3)
Milk	0	not detected	-	-
	2	2.65	132.5	1.03
	4	4.45	111.25	0.05
	6	5.2	86.7	2.13

## Data Availability

Not applicable.

## Supplementary Materials

The following supporting information can be downloaded at: https://www.mdpi.com/article/10.3390/bios13020242/s1, Figure S1: (a) The ultraviolet-visible spectra of the SnCl_2_.H_2_O precursor and SnSe-3MPA quantum dots. (b) Tauc’s plot to estimate the indirect band gap energy and (c) the excitation-emission photoluminescence spectra of the SnSe-3MPA quantum dots. (d) The proposed valance and conduction band structure of the SnSe(3-MPA) quantum dots. Figure S2: (a) The FT-IR spectrum of the Sn(3-MPA) (BLACK) complex and SnSe-3MPA quantum dots (RED); (b) the chemical presentation of the 3MPA-capped SnSe quantum dot nanomaterials. Figure S3: The square-wave responses of the ER-α/SnSe-3MPA/AuE biosensor corresponding to different concentrations of 17β-estradiol in 0.1 M phosphate buffer solution of pH 7.4 under aerobic conditions (i.e., expanded potential window). Figure S4: The square-wave responses of the ER-α/SnSe-3MPA)/AuE biosensor corresponding to different concentrations of 17β-estradiol in the absence of oxygen molecules, anaerobic conditions (i.e., expanded potential window). Figure S5: The cyclic voltammograms representing different precursor materials deposited on a gold electrode surface in 0.1 M phosphate buffer solution, pH 7.4, at a scan rate of 30 mV/s. References [65,66,67,68,69,70,71] are cited in the supplementary materials.
