# Supplementary material for: Quantum Dot-Sensitised Estrogen Receptor-α-Based Biosensor for 17β-Estradiol
Source: Biosensors (Basel). 2023 Feb 8;13(2):242. doi: 10.3390/bios13020242 (PMC9954354; doi:10.3390/bios13020242)
Supplement: Supplementary file 1 [file biosensors-13-00242-s001.zip › biosensors-2146802-supplementary.pdf]

Supplementary Material

Quantum Dot-Sensitised Estrogen Receptor-α-Based Biosensor for 17β-Estradiol

Abongile N. Jijana [1,2,*], Usisipho Feleni [3], Peter M. Ndangili [4], Mawethu Bilibana [5], Rachel F. Ajayi [2] and Emmanuel I. Iwuoha [2,*]

[1] Nanotechnology Innovation Centre, Advanced Materials Division, Mintek, Private bag X3015, Randburg, Johannesburg 2125, South Africa
[2] SensorLab (University of the Western Cape Sensor Laboratories), 4th Floor Chemical Sciences Building, University of the Western Cape, Private Bag X17, Bellville, Cape Town 7535, South Africa
[3] Institute for Nanotechnology and Water Sustainability (iNanoWS), College of Science, Engineering and Technology, University of South Africa, Florida Campus, P/Bag X6, Florida, Roodepoort, Johannesburg 1710, South Africa
[4] School of Chemistry and Material Science, The Technical University of Kenya, Nairobi P.O. Box 52428-00200, Kenya
[5] Department of Chemistry, School of Physical and Chemical Sciences, Faculty of Natural and Agricultural Sciences, North-West University (Mafikeng Campus), Private Bag X2046, Mmabatho 2735, South Africa
* Correspondence: abongilej@mintek.co.za (A.N.J.); eiwuoha@uwc.ac.za (E.I.I.); Tel: +27-(0)-11-709-4475 (A.N.J.); +27-(0)-21-959-3054 (E.I.I.)

1. Results and Discussion

1.1. Optical Properties of the 3-Mercaptopropionic Acid-Capped Tin Selenide Quantum Dots

The UV-Vis analysis of the SnSe-3MPA quantum dots shown in **Figure S1**a demonstrates a sharp and intense band at λ_{max} of 238 nm and a quantum dot-size-dependent band at λ_{max} of 285 nm. The high-intensity absorbance energy band around the λ_{max} of 238 nm in the spectrum of the $SnCl_2.2H_2O$ was also observed in the spectra of the SnSe-3MPA quantum dots. However, a blue shift in the absorbance from 238 nm (for $SnCl_2.2H_2O$) to 235 nm (for SnSe-3MPA quantum dots) was observed, supplemented by a drop in the absorbance from 2.6 to 0.98 for SnSe-3MPA quantum dots. This indicates confirmation of the chemical structural changes of $SnCl_2$ to SnSe-3MPA. The electronic band structure of $SnCl_2$ constitutes electronic transitions that result from metal to ligand charge transfer bands due to the presence of electron pairs of the tin metal and chlorine ligands. Charge transfer bands occur in complexes due to the presence of metals and ligands that either have donor or acceptor properties or exhibit both [65].

(a)

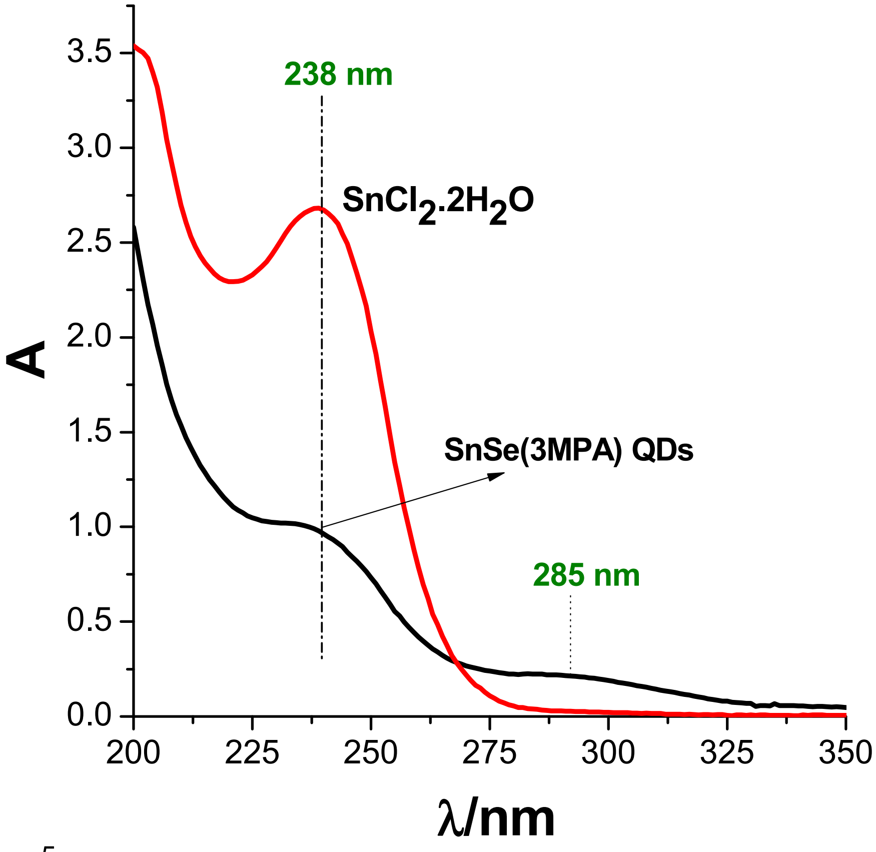

238 nm
SnCl2.2H2O
SnSe(3MPA) QDs
285 nm
A
λ/nm

(b)

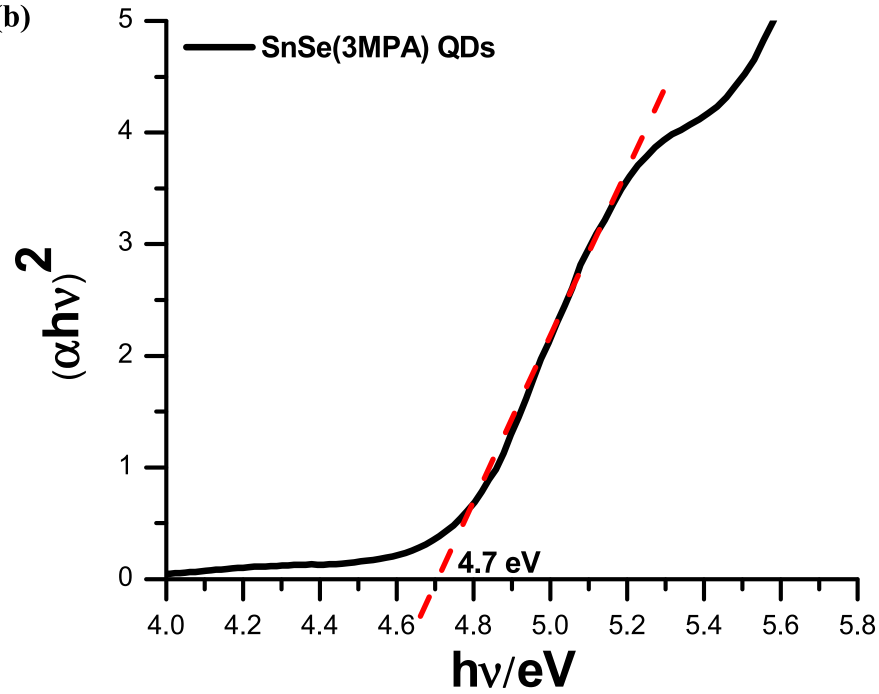

SnSe(3MPA) QDs
4.7 eV
(αhν)2
hν/eV

(c)

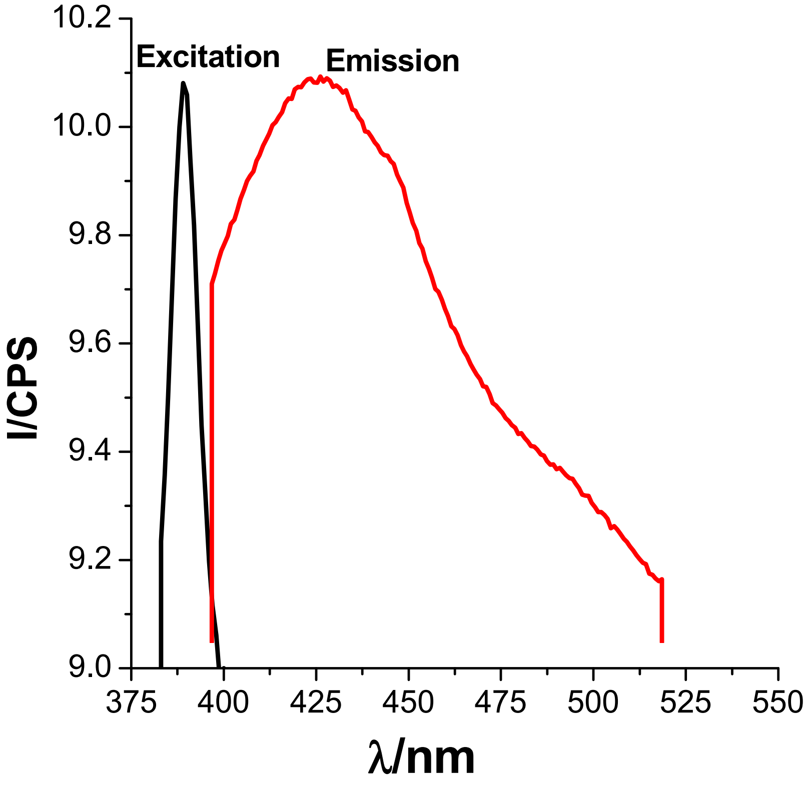

(d)

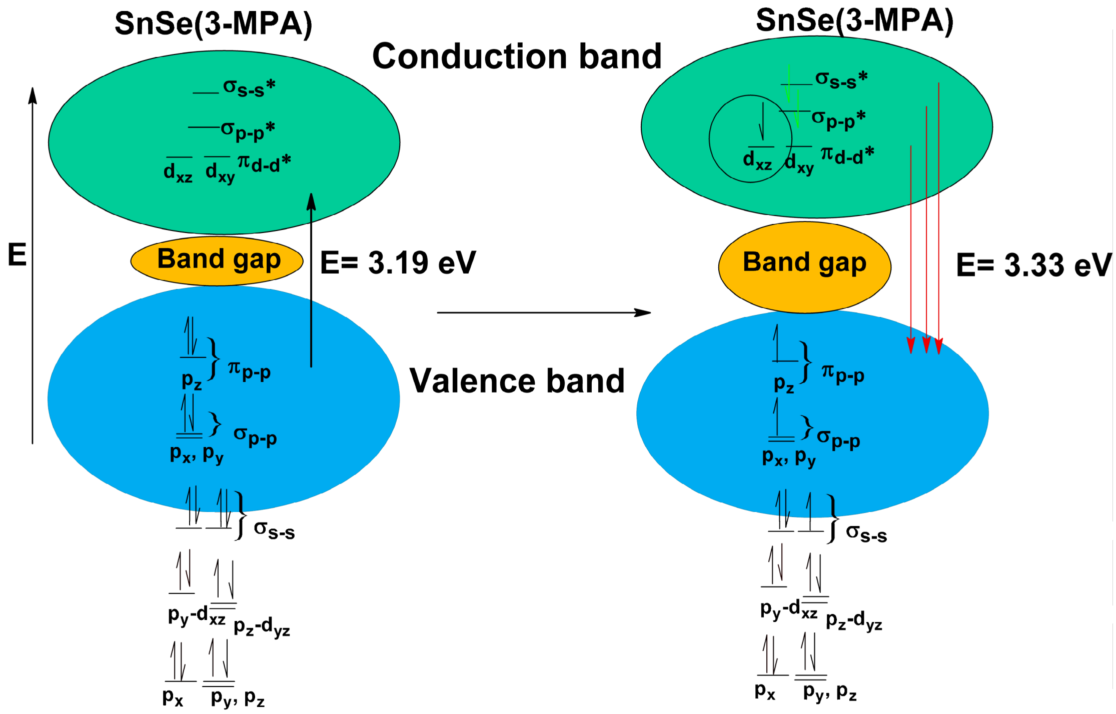

Figure S1. (a) The ultraviolet-visible spectra of the $SnCl_2.H_2O$ precursor and SnSe-3MPA quantum dots. (b) Tauc's plot to estimate the indirect band gap energy and (c) the excitation-emission photoluminescence spectra of the SnSe-3MPA quantum dots. (d) The proposed valance and conduction band structure of the SnSe-3MPA quantum dots.

The UV-Vis maxima absorbance band observed at 285 nm for the SnSe-3MPA quantum dots in **Figure S1** (a) was suggested to be due to the electronic transitions from the

valance band to the conduction band of the quantum dots [66]. This allowed for the determination of the optical indirect band gap energy of the SnSe-3MPA quantum dots using Tauc's photon energy estimation, which relates the quantum dots' optical properties with their photon energy properties. The corresponding estimation of the indirect band gap energy is presented in **Figure S1** (d). In Tauc's equation [67], the absorption coefficient α and photon energy (hν) are related to the band gap energy (E_g), as shown in Equation 1. The parameter h is the Planck constant, c is the speed of light, ν is the frequency of the absorbed photons, and the x-intercept designates the indirect band gap of a semiconductor or conducting material.

$$\alpha h\nu = c(h\nu - E_g)^{1/2} \quad \text{Equation 1}$$

The band gap of the SnSe-3MPA quantum dots was estimated to be 4.17 eV and within the wide band gap semiconductor range.

The photoluminescence properties of the quantum dot chromophores occur due to the electron-hole pair recombination phenomenon [68], which induces photon energy electronic transitions between their ground state-valance energy bands and the exited state-conduction bands. A proposed electronic band structure of the SnSe-3MPA quantum dots is illustrated in **Figure S1** (d). Fluorescence was used to evaluate the excitation and emission properties of the SnSe-3MPA quantum dots at the fluorescence wavelength region between 300 and 575 nm. The spectra showing the excitation and emission profiles of the SnSe-3MPA quantum dots are illustrated in Figure S1 (c). The excitation fluorescence spectrum exhibits a maximal excitation peak ($\lambda_{excitation\text{-}max}$) at 383 nm associated with the $\pi_{p\text{-}p}\rightarrow\pi^*,\sigma^*$ electronic transitions. Its energy gap was determined to be 3.19 eV, estimated using photon energy conversion. The corresponding emission peak ($\lambda_{emission\text{-}max}$) at 425 nm was associated with the energy separation gap of 3.33 eV due to $\pi^*,\sigma^*\rightarrow\pi_{p\text{-}p}$ electronic transitions. The SnSe-3MPA quantum dots were highly photo-luminescent due to electronic energy transitions between the valence and conduction bands, associated with the π^* to π (i.e., $\pi^*_{d\text{-}d}\rightarrow\pi_{p\text{-}p}$) electronic transitions. The narrowing of the band gap energy (i.e., the energy difference between conduction and valence bands) alludes to incomplete recombination of the electrons with the corresponding holes due to the presence of defects [68].

1.2. Chemical Structure Properties of the SnSe-3MPA Quantum Dot Nanomaterials

Figure S2 (a) illustrates the *FT-IR* spectrums of the Sn^{2+}(3-MPA) complex precursor and the SnSe-3MPA quantum dots. The vibrational frequencies at 2114, 1660, 1394, 1275, and 780 cm^{-1} were assigned to functional groups 3-8, as denoted in **Figure S2** (b). The chemical functional groups 3-8 are further ascribed to the vibrational frequencies ν(C-O), $\nu(C{=}O)_{strong}$, $\nu(C\text{-}H)_{bending\ mode}$, ν(C-O) [69,70] and ν(Sn-S) (i.e., a metal-sulphide bond [71]), respectively.

An increase in the transmission percentage ratio (C-O):ν(C=O) ($I_{C\text{-}O}/I_{C=O}$) for the Sn(3-MPA) complex and SnSe-3MPA quantum dots ranging from 0.04 to 0.98 was observed at vibrational band frequencies of 2114 and 1660 cm^{-1}, respectively. An increase in the $I_{C\text{-}O}/I_{C=O}$ ratio might indicate a change in the plane of symmetry adopted by a molecule or a bond. The C=O and C-O bond vibrational modes were somehow distorted to some degree in the presence of selenide ions due to obvious steric hindrance. This phenomenon is explained by Figure S2 (b). The steric effect/ hindrance feature in the presence of the selenide anion was further supported by the complete disappearance of or reduction in the intensity of the ν(C-H) vibrational and bending mode frequencies observed at 1394 and 1293 cm^{-1}.

(a)

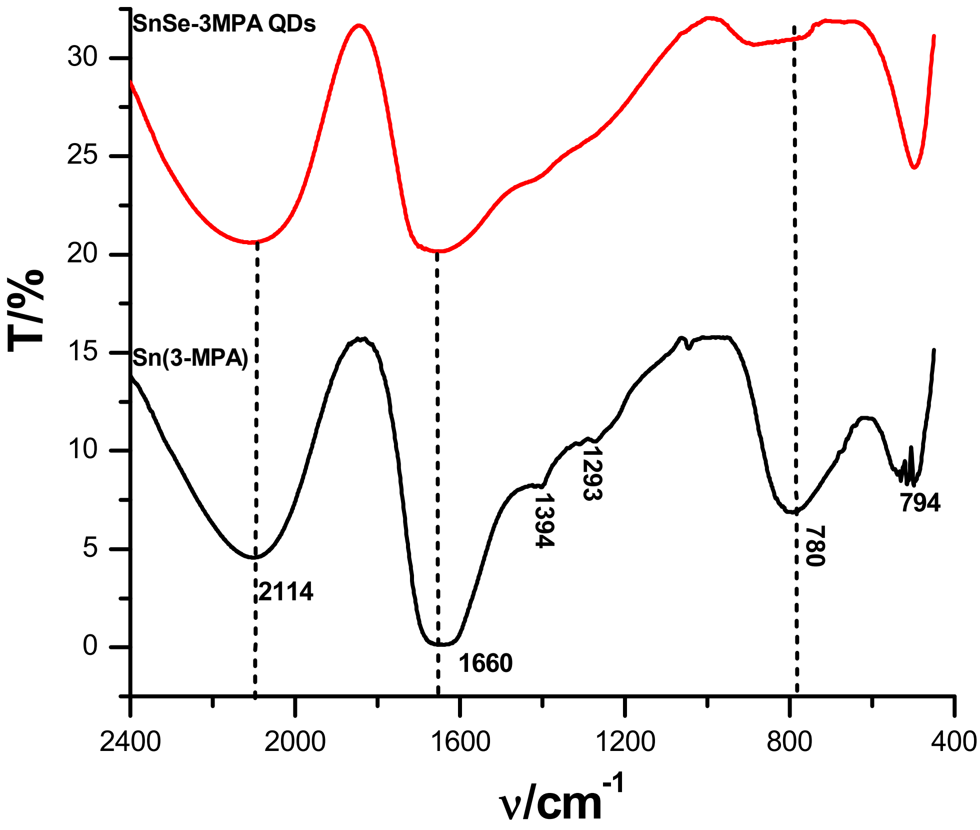

(b)

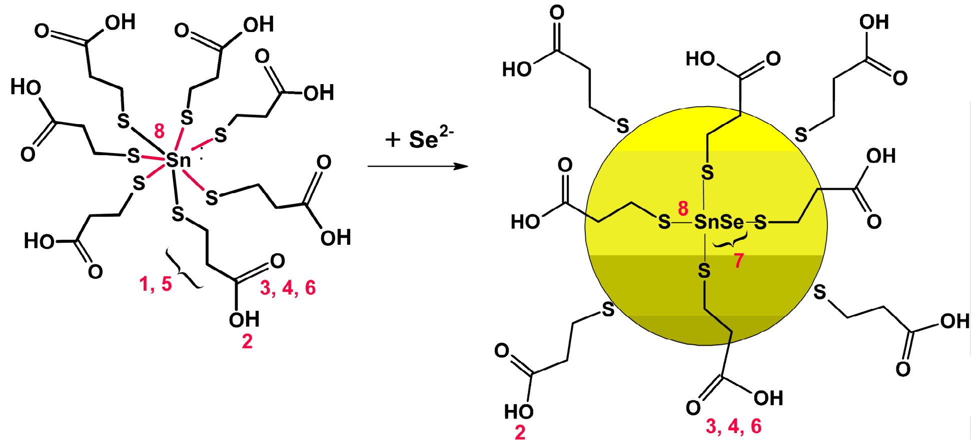

Figure S2. (a) The FT-IR spectrum of the Sn(3-MPA) (BLACK) complex and SnSe-3MPA quantum dots (RED). (b) The chemical presentation of the 3MPA-capped SnSe quantum dot nanomaterials.

A significant decrease in the transmission percentage at the IR frequency of 780 cm^{-1}, which is ascribed to ν(Sn-S), can be indicative of a significant reduction of the in-plane Sn-S bonds, hence, rectifying the formation of predominantly Sn-Se bonds. The 3-mercapto-propionic acid-capping ligand functionalization around the surface quantum dots was further confirmed by the presence of aliphatic bonds and carboxylate ion-related functional groups on the IR spectrum of the SnSe-3-MPA quantum dots. The chemical structure mapping of the 3MPA-capped SnSe quantum using FT-IR allowed for the prediction of the configuration of the capping ligands around the SnSe surface in the manner alluded to in **Figure S2.**

1.3. *Electrochemical Properties of the ER-α/SnSe-3MPA/AuE Receptorsensor*

The electro-analytic behaviour of the ER-α/SnSe-3MPA/AuE biosensor was assessed in the presence of different concentrations of 17β-estradiol in 0.1M phosphate buffer solution of pH 7.4 saturated with oxygen molecules **(aerobic conditions).** A scan rate of 30 mV/s was used. The resultant square-wave voltammograms are shown in **Figure S3**.

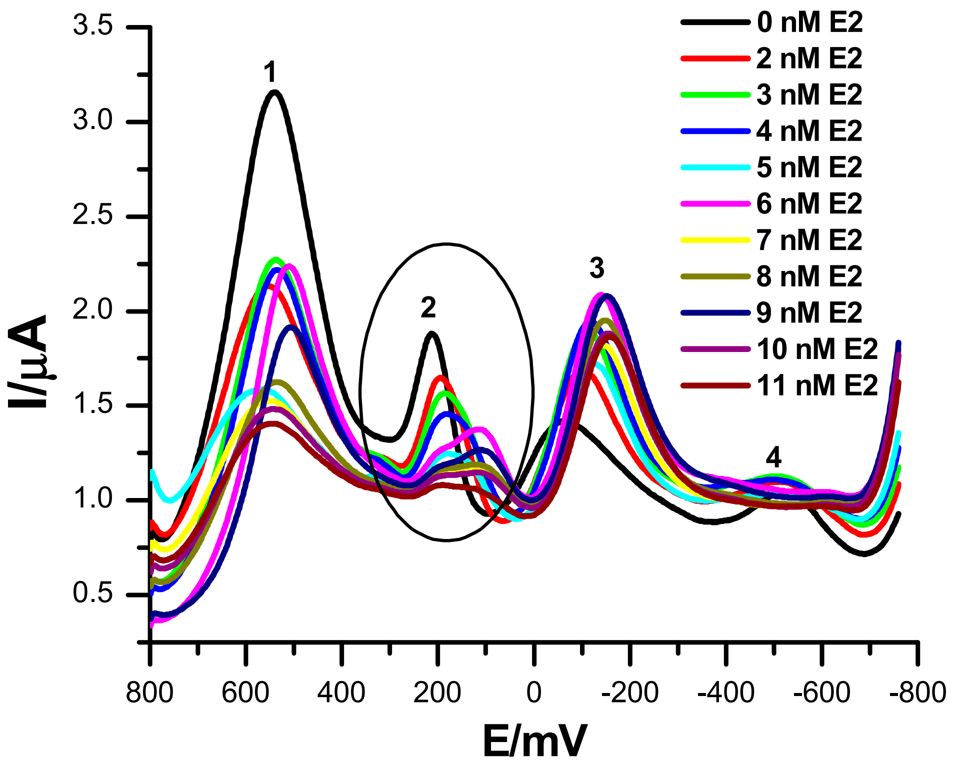

Figure S3. The square-wave responses of the ER-α/SnSe-3MPA/AuE biosensor corresponding to different concentrations of 17β-estradiol in 0.1 M phosphate buffer solution of pH 7.4 under **aerobic conditions** (expanded potential window).

Based on the voltammograms presented in **Figure S3**, the ER-α/SnSe-3MPA/AuE biosensor showed multiple redox reactions at peaks **1**, **2** and **3** and **4** at zero concentration of the 17β-estradiol analyte in the presence of dissolved oxygen molecules. The redox peaks were observed at the formal peak potentials ($E^{0'}$) values of 540 mV, 214 mV, -149 mV, and 514 mV. A successive increase in the 17β-estradiol concentrations influenced no progressive catalytic activity at the redox peaks **1, 3,** and **4**. This is attributable to un-specific interactions occurring at alternative binding sites/domains of ER-α. Pertinent to the observations, the electrochemical-catalytic effect corresponding to a gradual increase in the 17β-estradiol concentration was solely observed at a formal **peak potential 2** (i.e., at an $E_p^{o'}$ of 212 mV).

Figure S4 next shows the full range of square-wave voltammetry responses of the biosensor at successive additions of 17β-estradiol in a phosphate buffer solution of pH 7.4 at the scan rate of 30 mV/s in the absence of dissolved oxygen species (**anaerobic conditions**). This was achieved by purging the electrolyte solution with argon gas prior to and during the electro-analytical measurements. The voltammograms exhibited a well-pronounced reduction **peak (1) at $E^{0'}$ = 216 ±12 mV** after successive additions of 17β-estradiol. This is attributable to the electro-catalytic reduction and interaction of the 17β-estradiol with the ER-α modified on the biosensor surface. Moreover, an unspecific binding occurrence was observed at redox peaks labelled 2, 3 and 4.

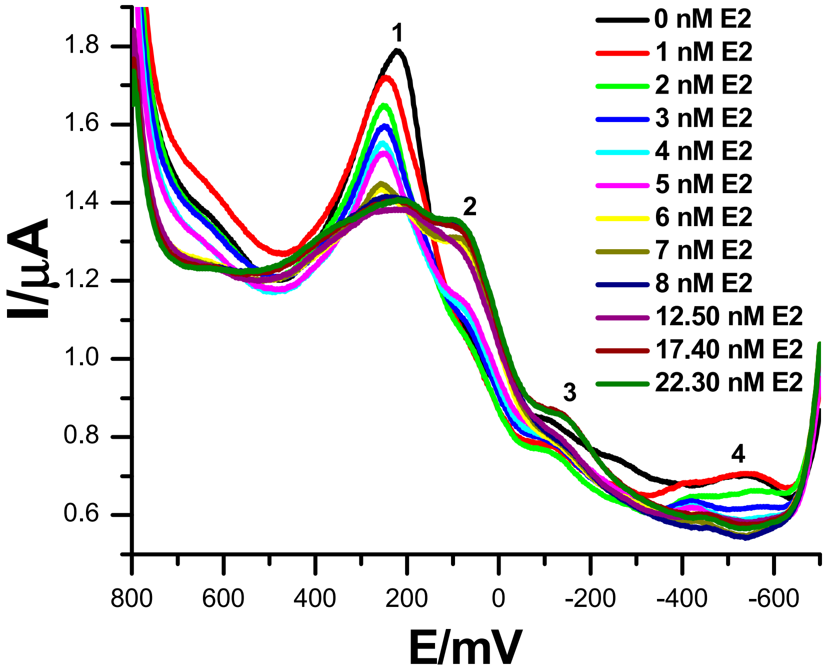

Figure S4. The square-wave responses of the ER-α/SnSe-3MPA/AuE biosensor corresponding to different concentrations of 17β-estradiol in the absence of oxygen molecules**, anaerobic conditions** (i.e., expanded potential window).

1.4. Electrochemical Properties of Different Precursor Materials Employed During the Synthesis of SnSe-3MPA

The cyclic voltammograms shown in **Figure S5** (a) to (2) represent 2 µL of the (a) bare AuE, (b) NaHSe, (c) $SnCl2._2H_2O$, and (d) 3MPA precursors deposited on the gold electrode. The electrochemical properties of the materials were evaluated at a potential window between 800 and -800 mV. A scan rate of 30 mV/s and 0.1 M of a phosphate buffer solution (electrolyte) of pH 7 were used. This investigation was done to establish the voltammetry electro-active components contributing to the overall electrochemical behaviour of the SnSe-3MPA quantum dots.

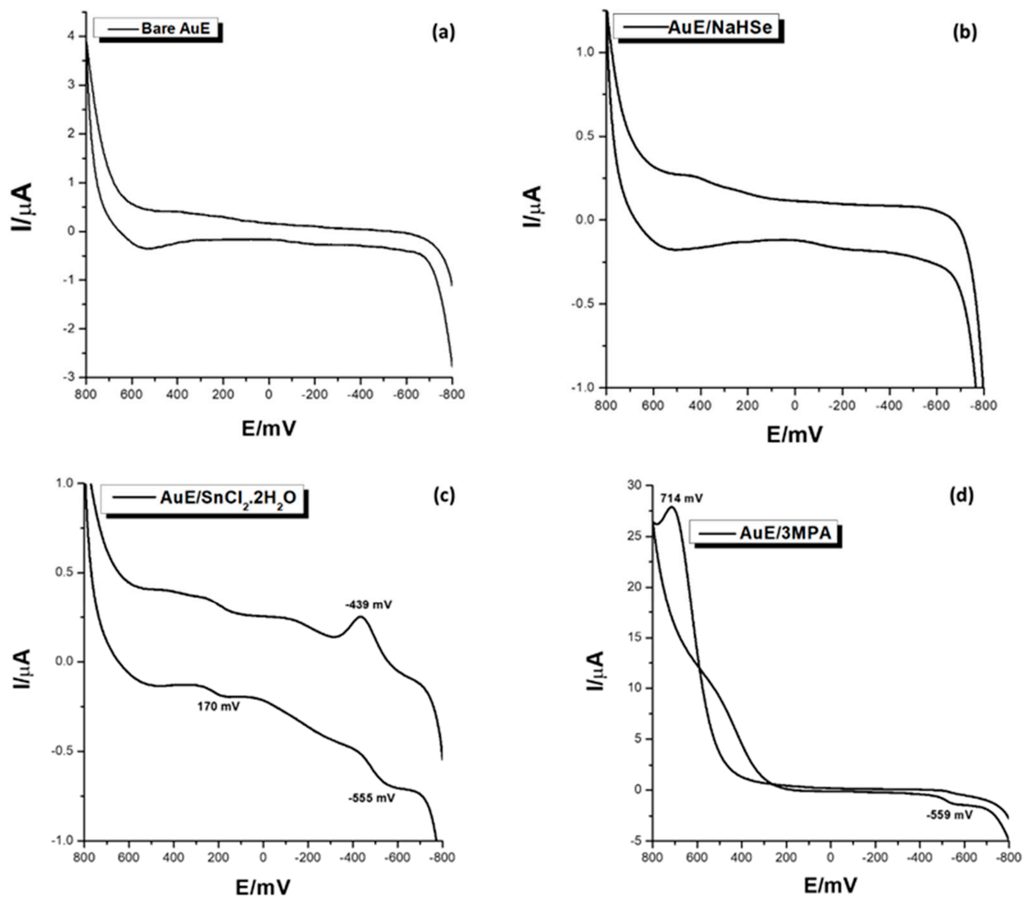

Figure S5. The cyclic voltammograms representing different precursor materials deposited on a gold electrode surface in 0.1 M phosphate buffer solution, pH 7.4, at a scan rate of 30 mV/s.

As shown in the cyclic voltammograms of **Figure S5** (a) and (b), the bare gold electrode and NaHSe precursors did not result in any redox peaks. However, upon modification of the gold electrode with $SnCl_2$ {Figure S5 (c)}, a distinct reduction peak with the peak potential Ep = -439 mV was observed. Subsequently, two other reduction peaks were observed during the cathodic scan, appearing at Ep = 170 mV and -555 mV. The capping ligand of 3-mercaptopropionic acid (3-MPA) (**Figure S5** (d)) also showed a bit of electrochemical activity, characterised by oxidation and reduction peaks at Ep = 714 and 559 mV, respectively.
